# Architectural frameworks: defining the structures for implementing learning health systems

**DOI:** 10.1186/s13012-017-0607-7

**Published:** 2017-06-23

**Authors:** Lysanne Lessard, Wojtek Michalowski, Michael Fung-Kee-Fung, Lori Jones, Agnes Grudniewicz

**Affiliations:** 1 Telfer School of Management, University of Ottawa, 55 Ave. Laurier E, Ottawa, ON K1N 6N5 Canada; 2 Institut du Savoir Montfort (ISM), 202-745A Montreal Road, Ottawa, ON K1K 0T1 Canada; 3 Departments of Obstetrics-Gynecology and Surgery, Faculty of Medicine, University of Ottawa, 451 Smyth Rd, Ottawa, ON K1H 8M5 Canada; 4 The Ottawa Hospital–General Campus, University of Ottawa/Ottawa Regional Cancer Centre, 501 Smyth Rd, Ottawa, ON K1H 8L6 Canada; 5 University of Ottawa, 55 Ave. Laurier E, Ottawa, ON K1N 6N5 Canada

**Keywords:** Learning health system, Architectural framework, Pre-implementation, Decision-support tools

## Abstract

**Background:**

The vision of transforming health systems into *learning* health systems (LHSs) that rapidly and continuously transform knowledge into improved health outcomes at lower cost is generating increased interest in government agencies, health organizations, and health research communities. While existing initiatives demonstrate that different approaches can succeed in making the LHS vision a reality, they are too varied in their goals, focus, and scale to be reproduced without undue effort. Indeed, the structures necessary to effectively design and implement LHSs on a larger scale are lacking. In this paper, we propose the use of architectural frameworks to develop LHSs that adhere to a recognized vision while being adapted to their specific organizational context. Architectural frameworks are high-level descriptions of an organization as a system; they capture the structure of its main components at varied levels, the interrelationships among these components, and the principles that guide their evolution. Because these frameworks support the analysis of LHSs and allow their outcomes to be simulated, they act as pre-implementation decision-support tools that identify potential barriers and enablers of system development. They thus increase the chances of successful LHS deployment.

**Discussion:**

We present an architectural framework for LHSs that incorporates five dimensions—goals, scientific, social, technical, and ethical—commonly found in the LHS literature. The proposed architectural framework is comprised of six decision layers that model these dimensions. The performance layer models goals, the scientific layer models the scientific dimension, the organizational layer models the social dimension, the data layer and information technology layer model the technical dimension, and the ethics and security layer models the ethical dimension. We describe the types of decisions that must be made within each layer and identify methods to support decision-making.

**Conclusion:**

In this paper, we outline a high-level architectural framework grounded in conceptual and empirical LHS literature. Applying this architectural framework can guide the development and implementation of new LHSs and the evolution of existing ones, as it allows for clear and critical understanding of the types of decisions that underlie LHS operations. Further research is required to assess and refine its generalizability and methods.

## Background

Since the early 2000s, increased attention has focused on understanding, designing, and implementing learning health systems (LHSs) as a means to improve the quality, responsiveness, efficiency, and effectiveness of healthcare delivery. While various definitions of the LHS exist in the literature, the most authoritative source is the Institute of Medicine (IOM), which envisions “the development of a *continuously learning health system* in which science, informatics, incentives, and culture are aligned for continuous improvement and innovation, with best practices seamlessly embedded in the delivery process and new knowledge captured as an integral by-product of the delivery experience” [1]. The current operationalization of various LHS initiatives also demonstrates the important role that patient data plays in supporting continuous learning, improving decision-making [2–5], informing new research directions [6], and creating more efficient, effective, and safe systems [7].

A LHS strives to accelerate the generation and uptake of knowledge to support the provision of quality, cost-effective healthcare that improves patient outcomes [1, 3, 8, 9]. A LHS can be understood as a rapid-learning organizational system that quickly adapts to new clinical and research information about personalized treatments that are best for each patient and then supports the effective delivery of these treatments [10]. As such, LHSs incorporate continuous learning at the system, organizational, departmental, and individual levels, in cycles or loops moving from data to knowledge and then from knowledge to practice and back again. In single learning loops, information and feedback circulate to support the evaluation, management, and improvement of patient care [11, 12]. Dynamic LHS models are based on double-loop learning, whereby long-held assumptions about system-level values, norms, and policies are also challenged by questioning existing processes and procedures [13, 14]. However, in order to achieve truly continuous learning, it is necessary to arrive at triple-loop learning, wherein people understand the process by which they learn, and thus learn how to learn [15].

**Fig. 1:**
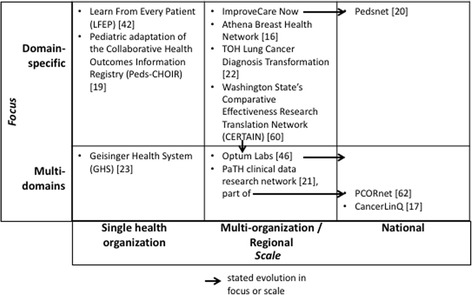
Sample of current LHSs initiatives categorized by focus and scale [16, 17, 19–23, 42, 46, 60, 62]

The IOM position paper and seminal literature primarily address national, all-encompassing LHSs. However, several recently proposed or adopted LHSs [16–21] show that these initiatives may vary in focus from domain specific (i.e., specific to a particular disease or concern such as lung cancer [22]) to multi-domain (i.e., spanning multiple diseases, for example, idiopathic pulmonary fibrosis, atrial fibrillation, and obesity [21]). They may also vary in scale, from a single healthcare organization, to multiple sites (often within a specific region or catchment area), to the national level. In Fig. 1, we categorize a sample of LHS initiatives to illustrate this variety. A given LHS may evolve, or aim to evolve, from domain specific to multi-domain, or from a local or regional scale to a national one.

While the initiatives summarized in Fig. 1 reflect the IOM’s high-level LHS vision and goals, their respective designs are actually predicated on very specific sets of assumptions, needs, purposes, core elements, and decisions. This highlights the fact that there are multiple ways in which the IOM vision for LHSs can be implemented. However, their specificity renders them unsuitable for reproduction in other contexts [2]. Indeed, simply using the elements of existing LHSs to guide the development and deployment of a new LHS hinders thinking about alternative choices that may better achieve the goals of the people involved in designing and implementing it. These include physicians, administrators, information technology specialists, and patients, broadly referred to as stakeholders in the remainder of the article.

Higher level operational frameworks are emerging from the deployment of LHSs and the lessons learned therein [23]. Nevertheless, they do not yet provide support for the numerous decisions to be made when developing and implementing a LHS, for example, which measures should be used to assess the achievement of a LHS’s goals, or which governance model is most appropriate for a given LHS. Moreover, despite existing contributions towards the articulation of LHS architectures [24, 25], a standardized approach incorporating all LHS components has yet to be created. To address this gap, we propose a comprehensive LHS architectural framework based on the well-recognized Federal Enterprise Architecture Framework [26]. The proposed LHS architectural framework allows the definition of the structures necessary for implementing a LHS in a manner that promotes rapid and systematic integration of evidence-based knowledge [27]. Such tools provide pre-implementation decision-support that helps to identify potential barriers and thereby increase the chances of successful implementation [28]. They also address the need to balance standardization and adaptation to local contexts, a classic challenge in implementation science.

### Our approach

A well-recognized approach for describing the structure and behavior of complex systems such as LHSs starts with creating their architectural framework. Such frameworks provide a holistic view of an organization as a system; they thus capture the structure of its main components at varied levels (including organizational and technical), the interrelationships among these components, and the principles that guide their evolution [28–30]. An architectural framework is not a detailed system design but rather a high-level blueprint of the system’s essential characteristics. Once an architectural framework has been defined for a particular type of system (such as LHSs), it can be used to guide the detailed design of specific systems (such as given LHSs each with its own context, purpose, etc.) through the development of models relevant to each component of the architectural framework. For example, business process models are commonly used to represent the core activities of an organization.

Using an architectural framework could guide people in a given health system context (for example, a group of physicians and nurses specialized in lung cancer care) to design, develop, and implement a LHS that both adheres to the larger LHS vision as defined by the IOM [1] and is adapted to their specific context. By using the framework, stakeholders can identify the various decisions that need to be made at each layer of a LHS, as well as the impacts of these decisions on the overall system. This would help to ensure that the resulting LHS initiative is context specific and fully addresses purposes, needs, and goals. An architectural framework also supports the evolution of a LHS over time by offering a consistent framework within which stakeholders review the alignment of goals and objectives, the adaptation of the system to its changing environment, and the adjustment of processes and functions [12]. Using a consistent architectural framework addresses a LHS’s need to orchestrate human, organizational, procedural, data, and information technology components [22]. A number of architectural frameworks outside the healthcare domain exist, each reflecting the context in which it was developed and the purpose for which it was created [29]. The most commonly used are the Zachman framework for information systems [25], the Open Group Architecture Framework for enterprise information technology [31], and the Federal Enterprise Architecture Framework [26]. The Zachman framework stands as an organizational ontology, describing an organization’s levels with basic questions such as “why,” “who,” “how,” etc. The Open Group Architecture Framework for enterprise information technology, as its name implies, captures an organization’s information technology components. The Federal Enterprise Architecture Framework captures an organization’s or system’s human and technical components. The latter is the most complete, combining the characteristics of the other two frameworks and enabling the alignment of multi-stakeholder goals within an organization’s structure and technical systems [32]. The Federal Enterprise Architecture Framework thus provides an ideal basis for LHS architectures situated in multi-professional health systems, such as hospitals or health maintenance organizations.

The Federal Enterprise Architecture Framework has been used to provide a standardized manner in which to capture US government agencies’ IT and organizational resources so as to enable resource sharing and prevent duplication. Numerous organizations outside of government settings have since applied it for the same purpose [32]. As such, it has been widely used and assessed through case studies and conceptual analysis [32, 33]. An investigation of its use within the US government has shown that its large scope of application and the obligation for agencies of varied nature to strictly comply to its guidelines has created issues for its users and limited its potential benefits [33]. These limitations can however be addressed by ensuring that an architectural framework is understood and used as a means to inform, guide, and constrain decisions, rather than become an end goal [33]. Accordingly, our approach to LHSs’ architectures addresses this challenge by promoting the use of the framework as a flexible, context-specific guide rather than as a prescriptive standard.

The Federal Enterprise Architecture Framework includes the architectural framework itself, named the Consolidated Reference Model, and an accompanying methodology. We focus here on the Consolidated Reference Model, which deconstructs an organization in terms of six decision layers: strategy, business, data, applications, infrastructure, and security. It recommends particular tools and artifacts within each layer to enable designers to develop more detailed plans within them such as, for example, process models within the business layer. It also provides recommendations for ensuring that each layer’s models are developed in harmony with the other layers. A data model, for example, should capture the information needed to measure the achievement of goals identified in the performance layer. We propose here to adapt the Consolidated Reference Model such that it captures LHS dimensions identified in the literature. This results in a high-level framework that is both grounded in practice and able to address the challenge of implementing new LHSs across different contexts, foci, and scales. It also guides the evolution of existing LHSs.

## Discussion

Here, we identify the five dimensions common to LHS in extant literature and then discuss the proposed architectural framework that captures these dimensions as an adaptation of the Consolidated Reference Model.

### Dimensions of learning health systems

Seminal literature [7, 12, 34, 35] and existing LHS initiatives [16, 19, 20, 36] reveal five key dimensions that capture the nature of a LHS. Core elements within each dimension point to decisions that must be made as LHSs are developed and implemented.

The first dimension captures the *goals* pursued by a LHS. These vary from better decision support at the point-of-care to continuous quality improvement. The next three dimensions, which form the core of a well-functioning LHS [36], derive from the Collaborative Chronic Care Network (C3N) platform [37, 38]: the *social dimension* focuses on building a community; the *technical dimension* addresses data integration; and the *scientific dimension* enables learning, innovation, and discovery [37]. The fifth dimension—ethics—is critical for ensuring that a LHS pursues its learning and innovation activities in a manner that protects patients’ rights and privacy [39].

#### Goals dimension

The overarching goal driving the LHS vision is to provide cost-effective, safe, and high-quality care, leading to the improvement of patient health and other outcomes [1, 7]. The IOM has called for 90% of clinical decisions to be supported by timely and up-to-date clinical information and to reflect the best available evidence by 2020 [2]. To achieve this objective, LHSs should create virtuous circles of continuous learning and improvement through data sharing and the generation of evidence-based knowledge [6, 40]. Since LHSs aim to increase the speed by which research is translated into improved patient care, they are sometimes referred to as “rapid-learning health systems” [9]. Accordingly, a LHS should be able to accelerate all elements of the knowledge generation and adoption process, including the introduction of new drugs, comparative effectiveness research, discovery and implementation of best practices, and patient and physician decision support for shared decision-making [10].

Existing LHS initiatives tend to focus on some of these factors. For example, some LHSs concentrate on patient and physician decision support (e.g., Peds-CHOIR [19]), while others focus on conducting studies that enable improved pathways, practices, and guidelines over time (e.g., PEDSnet [20]). A LHS may pursue a number of different goals, such as the Athena Breast Health Network initiative that seeks automated identification of at-risk patients, standardization in pathology reporting and recommendation practices, and improvement of care practices [16]. No existing LHS, as far as we are aware, aims to achieve all identified LHS goals. While this does not diminish the important and tangible benefits that existing LHSs provide, it does highlight the need to allow each LHS to pursue its own set of goals in line with its particular focus, scale, and evolution.

#### Social dimension

The networks of people and institutions that constitute a LHS must be considered as an integral component of that system, not just as passive users of its digital infrastructure [3]. An appropriate culture of transparency, collaboration and teamwork, innovation, and continuous learning must exist [15, 41, 42]. Elements necessary to generate such a culture include governance and leadership principles, appropriate decision-making processes, alignment of stakeholder goals (patients, clinicians, administrators, researchers), and requisite expertise (including clinical and analytic) [6, 37]. The resulting social dimension can take different forms, such as a matrix organization with distributed responsibilities and multi-site teams (Athena Breast Health Network [16]); a community of learning engaged in various activities like monthly teleconferences and learning sessions (PEDSnet [20]); or a centralized service model wherein one stakeholder is responsible for data analysis, quality improvement and assurance, and care coordination services (CancerLinQ [17]).

#### Technical dimension

The digital infrastructure supporting a LHS is critical to empowering the social dimension by driving innovation across the healthcare ecosystem. It is, therefore, essential for the successful fulfillment of LHS goals [3]. At the heart of this infrastructure lies reliable and analyzable health data [6]. Current LHSs show that data can originate from various sources, including electronic health records (EHR), patient entries, and associated healthcare information systems; registries using pre-specified and system-specific data fields filled by physicians; or surveys given to consenting patients participating in a research project. Although these sources can be integrated, existing domain-specific LHSs tend to use focused data repositories (e.g., LFPE [42], Peds-CHOIR [19]), while multi-domain LHSs are more likely to use full-EHR entries (e.g., CancerLinQ [17], PaTH [21]). While an infrastructure could be created for a specific source and type of data, an extensible architectural framework should make use of recognized interoperable standards for defining, exchanging, and synchronizing healthcare data [43].

Another element within the technical dimension is data lifecycle management. The walk through a chain of evidence from data collection to data transformation and consumption can be a challenging task, given the diversity of stakeholders involved in each step [44]. The ways in which data objects and the end-to-end data lifecycle are handled will likewise vary according to the selected technical architecture. Seminal LHS literature promotes a distributed approach to data management to ensure the infrastructure’s resilience [7]. In this perspective, data management can be distributed at different points during its lifecycle. In a federated system, for example, data repositories remain under the control and at the location of the institution producing the data, although data queries can originate from any participating institution, which then handles its own analysis [40, 45]. Alternatively, data repositories may be distributed but the results of data queries centralized (e.g., PaTH [21]). A fully centralized approach to data management may be preferred to better handle real-time requests (e.g., Peds-NET [38]) or advanced analytics (e.g., Optum Labs [46]).

#### Scientific dimension

While learning may happen within any of the abovementioned LHS dimensions, it is the scientific dimension that most fosters learning by focusing on discovering and testing innovations for improved health outcomes [37]. This dimension brings together the social and technical dimensions of a LHS into a continuous learning circle that moves from data aggregation and analysis to interpretation and practice change. This leads to the generation of new data that can be integrated within the learning system [11]. Such a learning circle ideally integrates both data collected at point-of-care and the results of various types of studies, such as comparative effectiveness research, methodological research, and behavioral and policy research (Optum Labs [46]). However, existing initiatives show that improvements in clinical care can also come from operational-level learning, for example, learning how to significantly reduce delays in patient diagnostic processes using lean process methods [47]. Knowledge can likewise be disseminated at different speeds and to various stakeholders—depending on the LHS’ goals—to provide real-time decision support for patients and care providers or for long-term changes in care pathways.

#### Ethical dimension

The fifth LHS dimension identified in the literature is perhaps the most challenging: the need for a moral framework to guide all learning activities within a LHS. Developing such a framework confronts the distinction between clinical research and clinical practice in terms of ethics, since the use of identifiable patient data for continuous learning within a LHS—that may involve several health providers—is neither a recognized form of clinical research nor routine use for clinical practice such as physician-patient encounters. Integrating patient data collected at the point of care with population-based research data is thus difficult to accomplish given existing ethical guidelines regarding patient privacy and data security. There has been preliminary work in this area, including the proposition of an ethical framework to guide LHS learning activities [39] and an exploratory study identifying the ethical issues faced by healthcare organizations wanting to transition to a LHS [9]. However, more research is needed to reach consensus regarding which learning activities require oversight and how to determine the extent of such an oversight.

To date, few LHSs explicitly address the ethical dimension. One example that does is the Geisinger Health System, which, as part of Geisinger’s transformation into a LHS, developed institutional guidelines for navigating the differences and overlap between quality improvement and research [23]. These guidelines aim to ensure that the oversight regimen emphasizes the optimization of learning anywhere along this continuum. Another example is the CancerLinQ initiative [17], which has created guiding principles that promote the ethical management and use of data through data stewardship and protection (including secure de-identification of patient data), as well as transparency and accountability to patients, providers, and eligible stakeholders.

### Components of a LHS architectural framework

**Fig. 2:**
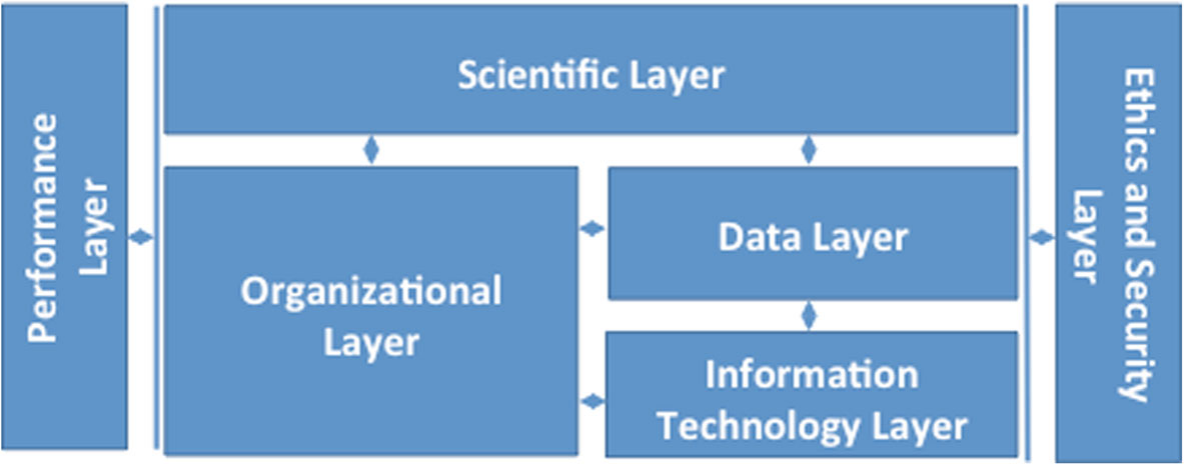
LHS architectural framework

We present here our proposed architectural framework that captures the five LHS dimensions discussed above. It is illustrated in Fig. 2.

Each decision layer in the proposed framework, except for the scientific layer, has been adapted from the Federal Enterprise Architecture Framework’s Consolidated Reference Model [33]. Table 1 provides a summary while the content and methods of each layer are described in more detail below.

**Table 1:** Overview of decision layers in the proposed architectural framework

Decision layer	Consolidated Reference Model	Role in the LHS architectural framework	Relevant LHS dimension
Performance	Prescribes priority and strategic goals, and measures to track goal achievement.	Prescribes goals taken from IOM Strategic Map	Goals
Scientific	N/A	Develops new transferable knowledge	Scientific
Organizational	Provides taxonomy with hierarchical description of the Federal Government in terms of sectors, business functions, and services.	Provides organizational taxonomy of a health system and its organizational units as well as its external stakeholders	Social
Data	Provides four domain taxonomies relating to mission, enterprise, guidance, and resource data.	Captures data sources for clinical and point-of-care data, and specifies data standards and lifecycle management procedures	Technical
Information technology	Categorizes applications and their components at three levels (systems, application components, and interfaces); categorizes information technology infrastructure components (platform, network, facility).	Brings together applications and infrastructure components given their varying importance across LHSs	Technical
Ethics and security	Defines security controls and measurements related to, e.g., regulatory conditions, risks, and compliance.	Adds ethical dimension related to privacy and security of patient data in line with existing legislative frameworks	Ethical

*N/A* not applicable

#### The performance layer

**Fig. 3:**
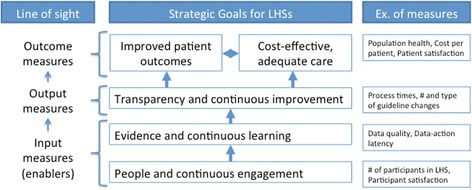
Categories of goals and possible measures in the performance layer

The performance layer identifies the goals pursued by a LHS, as well as measures to track the achievement of these goals. According to the IOM [1], LHSs have two strategic goals: better outcomes and lower costs. These goals are to be achieved through three components: people and continuous engagement, evidence and continuous learning, and transparency and continuous improvement. Figure 3 shows how the IOM’s strategic goals and components can serve as high-level goal categories for LHSs. It also shows how goals within each category can be related and measured to achieve the “line of sight” principle by which outcome measures are derived from output and input measures. Creating an architectural framework for a given LHS requires its stakeholders to state the specific goals that they wish to pursue within each category. Given the importance of patient engagement for LHS success, however, the component related to “people and continuous engagement” should always include goals related to engaging patients alongside physicians and other stakeholders. A LHS’s stakeholders should also agree upon the measures that will be used to assess the achievement of those goals.

A LHS that meets the IOM vision needs to emphasize social value, in particular contributing to a healthier population through better patient health outcomes alongside business value such as lower costs. The well-known balanced scorecard is one method that can be used to capture the goals and related measures of a LHS [48]. This method helps to identify the goals that are most important to an organization’s performance and then enables the organization to monitor their achievement and their impact on one another through a set of measures. It can also support double- and triple-loop learning. For example, if measures related to continuous learning and continuous improvements are satisfactory, but patient outcomes and cost measures deteriorate, the hypothesized causal relationships among these goals, the goals themselves, and even the structure of the learning process may need to be questioned.

#### The scientific layer

**Fig. 4:**
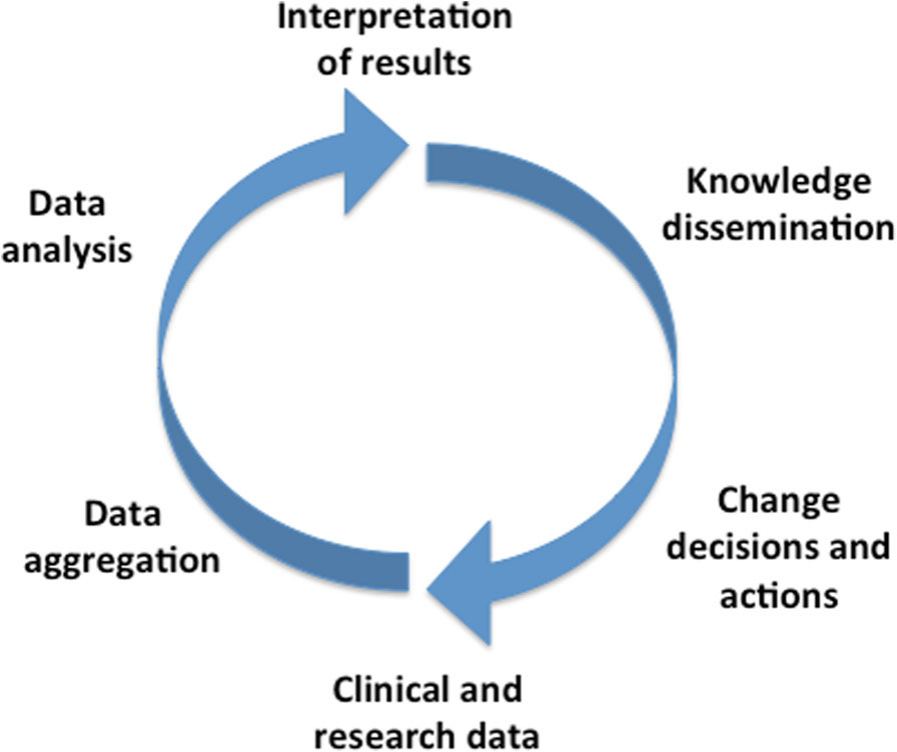
Generic learning cycle within the scientific layer

The scientific layer identifies the learning activities that will be undertaken in a given LHS, such as quality improvement or comparative effectiveness research. For each learning activity, a learning cycle needs to be defined that indicates which data should be collected, how they are to be analyzed, how data analysis will generate knowledge, what changes will be affected by this knowledge, and how those changes will be disseminated and implemented [11, 42]. See Fig. 4 (Generic learning cycle within the scientific layer). A learning cycle for comparative effectiveness research may thus differ from one seeking to improve best practices [49]. Given the dynamic and holistic nature of learning cycles, methods from systems theory (such as systems dynamics) can be used to model them in a formal manner [50]. This would serve to clarify where and how learning would happen in each activity. For example, learning from clinical data and systematic reviews may inform guidelines and pathways, while learning from patient management may inform process improvements. Given that different stakeholders and data may be required for each learning cycle, the scientific layer should drive decisions about organizational and data models.

#### The organizational layer

This layer of the architectural framework captures the chosen governance model and associated responsibilities. The methods used to formalize governance vary according to need. For example, a matrix structure may be described using well-known structured systems modeling notations, while communities of practice—groups of people engaging in collective learning about a domain of interest—may be better described using network models designed to highlight the relationships among individuals and organizations. Decision-making processes should also be identified and linked to the organizational structure. Given that knowledge-intensive processes, such as decision-making, require much more flexibility than routine ones, novel engineering methods may be needed to capture them [51]. Whichever method is adopted, it should explicitly capture how the LHS fosters patient engagement and where patients fit into the organizational structure and its decision-making processes. Moreover, the organizational layer should be aligned with the performance layer through, for example, the use of teamwork-linked performance measures and more general population-based health improvement measures.

#### The data layer

The data layer provides a common way to describe and share data across organizational boundaries. Two types of data may be used in a LHS: scientific (research) data and patient data collected at the point of care and in the population base. Since one cannot assume that relevant data is readily available, this layer identifies the LHS’s data sources. In the case where more than one data source is leveraged, this layer also needs to address interoperability issues [35], namely, the chosen recognized standards allowing for syntactic interoperability (the ability of systems to exchange data) such as Health Level Seven (HL7) [52], and controlled vocabularies and ontologies allowing for semantic interoperability (the ability to automatically interpret exchanged information) such as SNOMED (Systematized Nomenclature of Medicine) and RxNORM (a terminology that contains all medications available on the US market) [45, 53]. Data lifecycle management procedures and processes, including how data quality will be ensured [54] should also be defined in this layer. As such, the methods defined in this layer are strongly related to the framework’s ethics and security layer.

#### The information technology layer

The information technology layer enables a standardized manner of categorizing information and communication technology assets, whether software, hardware, or network related. Since the boundaries of a LHS may not align with organizational boundaries, the purpose of this layer is to address only those components that are used to store, analyze, and transform input data, and to disseminate results to LHS stakeholders. This layer should also capture how these assets are related, for example, through a centralized or decentralized model [55]. Given the likely presence of existing health information systems in institutions wanting to launch or transform themselves into a LHS, this layer needs to specify how information technology supporting a LHS interfaces with an institutions’ existing technology assets. Decisions made within this layer need to interface with other layers; a computer-supported tailored feedback tool, for example, must be related to a learning cycle in the scientific layer focused on clinical audit and feedback [56].

#### The ethics and security layer

This layer captures the ethical and privacy dimensions of health data collection and use as they relate to security controls and measures. As such, it should minimally encompass existing security and privacy legislative frameworks such as The Health Insurance Portability and Accountability Act of 1996 in the USA [57] or the Personal Information Protection and Electronic Documents Act and the Personal Health Information Protection Act in Canada [58, 59]. Moving forward, new frameworks encompassing guidelines for both clinical research and clinical practice [39] will likely be recognized and should be integrated. The choices made within ethics and security layer should further guide the development of scientific, data, and information technology procedures.

### Illustrating the use of the LHS architectural framework

While the architectural framework described here captures all five LHS dimensions through the decision layers, a given LHS may focus on only some of these layers. Taking contrasting examples from among those summarized in Fig. 1, we briefly describe two very different yet equally successful LHS initiatives using our proposed LHS architectural framework’s decision layers (see Table 2). A full application of the architectural framework would entail more comprehensive descriptions and the development of models within each layer. For now, Table 2 highlights the ability of the LHS architectural framework to capture very different initiatives in a common manner. As such, it facilitates an understanding of the choices that were made by each governance team.

**Table 2:** Illustration of the application of the proposed LHS architectural framework

Layer	Learn From Every Patient initiative [42]	PaTH clinical data research network initiative [21]
Performance	Goal: continuous quality improvement in clinical care for children with cerebral palsy. Measures: changes in healthcare utilization rates and related costs	Goal: informatics-supported infrastructure for cohort identification and data sharing for idiopathic pulmonary fibrosis, atrial fibrillation, and obesity. Measures: not discussed
Scientific	12-month study of one cohort, within a series of learning projects for continuous quality improvement	Comparative effectiveness randomized trials with specific research questions informed by clinical data research experts and vetted by informatics group
Organizational	Program team includes key clinical stakeholders, clinical and information technology teams	Steering Committee includes representatives from each site, three advisory committees (including patients), four working groups (research questions, information technology, methodology, regulations)
Data	Source: data fields and questions added to institution’s EHR. Data quality: ensured by database manager	Source: Complete set of longitudinal data about target populations taken from site EHRs. Standards: Standardized descriptions of data elements using established standards and vocabularies; use of HL7 by all participating health systems. Quality: manual and automated monitoring by data engineers.
Information technology	Existing EHRs and related infrastructure	Source data loaded onto centrally-maintained data warehouse; queriable through analytics interface
Ethics and security	No review board authorization required (all data collected appropriate for standard clinical care)	Not directly discussed; data transformation process includes de-identification prior to loading into warehouse

A methodology to guide a LHS’s implementation is needed to accompany the proposed LHS architectural framework. While a detailed discussion of such a methodology is beyond the scope of this paper, its general procedures might be drawn from the Collaborative Planning Methodology that accompanies the Consolidated Reference Model [33]. This is a five-step iterative process divided into two phases: organize and plan, and implement and measure. The first phase focuses on developing consensus among all stakeholders about the needs, purpose, governance system, and content of the LHS architectural framework. While the framework focuses on the system, the accompanying methodology focuses on guiding discussions among the people involved in designing and implementing a LHS. The methodology accompanying the use of the framework should thus ensure active stakeholder engagement throughout the design and implementation process. A number of reports on emerging and existing LHSs emphasize this point, for example, in terms of the importance of identifying the people and teams that should be involved [23, 42] and of mobilizing a healthcare community around a LHS initiative [16, 22, 60]. This is an important point, since individual and team learning are needed before institutional or system-level learning can occur in the form of improved processes, clinical guidelines, and more. Finally, the methodology does not assume a complete implementation, without planned future improvements. Rather, it accounts for progressive and iterative deployment and improvements as needed.

A smaller scale initiative may start by making decisions and developing models within the organizational and scientific layers, emphasizing the involvement and buy-in of key individuals and teams in one learning project that utilizes existing data and information technology assets. As the benefits of the first project become apparent, the governance team can use that momentum to identify other learning cycles, articulate goals and measures in the performance layer, address additional data storage and analysis needs, etc. In this approach, the LHS architectural framework serves to build learning capacity within the governance team at individual and group levels, which is key to implementation sustainability [61]. The stakeholders of a larger scale initiative may have the necessary resources and organizational support to address all layers of the LHS architectural framework in its first iteration. Using the framework in this manner should ensure that alignment and learning happens across siloes, by supporting communication among clinical and administrative stakeholders, as well as information technology specialists.

## Conclusion

In this paper, we present an LHS architectural framework that captures common LHS dimensions identified in the literature and inspired by the Federal Enterprise Architecture Framework’s Consolidated Reference Model [33]. We propose this framework as a high level yet practical means to guide the development, implementation, and evolution of LHSs. While exemplars highlight the possibility that existing approaches can successfully make the LHS vision a reality, their variability and the design implications that these variations entail mean that many of these approaches cannot be easily replicated. Applying the LHS architectural framework discussed here unifies the core components of LHSs while facilitating a better understanding of variations among systems and the types of decisions that these systems support. By enabling the analysis of existing LHS initiatives in a consistent manner, our proposed framework allows for reproduction, adaptation, and scaling of these initiatives. By supporting decision-making, our framework can support LHS implementation in the following manner:

- Performance layer: helps to identify, relate, and measure context-specific goals that can achieve the LHS vision.
- Scientific layer: guides the definition of learning cycles that are specific to desired learning outcomes while addressing common elements, such as how data are collected and analyzed, or how results are implemented.
- Organizational layer: helps to capture a governance model and associated responsibilities.
- Data layer: supports the description of data used in the scientific layer, as well as processes to ensure their quality.
- Information technology layer: supports the categorization of the information technology assets used to store, analyze, and transform data in the data layer.
- Ethics and security layer: facilitates the capture of the ethical and privacy ramifications of health data collection and use as they relate to security controls and privacy legislative measures.

Future research is required to assess and further refine the generalizability and methods used in the proposed framework. Nevertheless, given the ineluctable emergence of new LHS initiatives, the guidance offered by LHS-specific architectural frameworks, such as the one presented in this paper, is critical for supporting repeatable and successful LHS implementation at varied scales and foci.
